# Prospective Clinical Evaluation of Customized Titanium Occlusive Barriers with Window Modification for Guided Bone Regeneration: Radiographic and Histological Outcomes

**DOI:** 10.3390/biomimetics11020149

**Published:** 2026-02-17

**Authors:** Luis Leiva-Gea, Alfonso Lendínez-Jurado, Paulino Sánchez-Palomino, Bendición Delgado-Ramos, María Daniela Corte-Torres, Cristina López-De La Torre, Isabel Leiva-Gea, Antonio Leiva-Gea

**Affiliations:** 1 Clínicas ClearDent, 23006 Jaén, Spain; leiva1987@hotmail.com; 2 Facultad de Odontología, Universidad de Murcia, 30003 Murcia, Spain; 3 Hospital Regional Universitario de Málaga, 29011 Málaga, Spain; 4 Facultad de Medicina, Universidad de Málaga, Andalucía Tech, Campus de Teatinos s/n, 29071 Málaga, Spain; 5 Instituto de Investigación Biomédica de Málaga (IBIMA)-Plataforma BIONAND, 29010 Málaga, Spain; antonioleivagea7@gmail.com; 6 Distrito Sanitario Málaga-Guadalhorce, 29009 Málaga, Spain; 7 Facultad de Odontología, Universidad de Granada, 18011 Granada, Spain; paulinosanchezp@gmail.com (P.S.-P.); bendi.delgado@gmail.com (B.D.-R.); 8 Biobanco del Principado de Asturias, FINBA-ISPA, 33011 Oviedo, Spain; mdanielac@hotmail.com; 9 Departamento de Biomedicina y Odontología, Facultad de Ciencias Biomédicas y Deporte, Universidad Europea de Andalucía, 29010 Málaga, Spain; cristina.lopez3@universidadeuropea.es; 10 Hospital Universitario Virgen de la Victoria, 29010 Málaga, Spain

**Keywords:** guided bone regeneration, alveolar ridge augmentation, customized titanium mesh, beta-tricalcium phosphate, digital workflow, dental implants

## Abstract

This study aimed to quantify horizontal and vertical bone gain using superimposition of preoperative and postoperative cone beam computed tomography (CBCT) in severe alveolar ridge defects treated with a modified guided bone regeneration (GBR) technique based on customized titanium occlusive barriers with a window design, combined with autologous blood clot and β-tricalcium phosphate (β-TCP). In this prospective case series, 13 patients (28 defects) were treated. Customized titanium barriers were digitally designed based on CBCT data and manufactured by laser sintering. The barriers were fixed over the defects and filled with a mixture of an autologous blood clot and β-TCP, providing an osteoconductive scaffold within a stable regenerative compartment. A standardized window-based follow-up protocol was applied during healing, including irrigation and controlled deepithelialization. Primary outcomes were horizontal and vertical bone gain, assessed by pre- and postoperative CBCT superimposition. Histological evaluation was performed at the time of implant placement. After 8 months, significant bone gain was observed, with a mean horizontal gain of 4.50 ± 2.02 mm and a mean vertical gain of 4.40 ± 2.82 mm (*p* < 0.0001), confirmed by linear mixed-effects models and patient-level sensitivity analyses (*p* < 0.001). Histological analysis revealed well-vascularized newly formed bone with active osteoblasts and no inflammatory response. Keratinized gingiva formation was observed at all sites. One minor complication (mild screw loosening) was recorded and successfully resolved. This study is presented as a prospective case series; therefore, the results should be interpreted as exploratory evidence and do not allow direct comparisons or conclusions regarding equivalence or superiority over other GBR techniques. The present report specifically evaluates the regenerative phase prior to functional loading; therefore, although implants were placed according to protocol, implant survival and long-term functional outcomes were not assessed and cannot be inferred from these data. Within the limitations of this prospective case series, customized titanium occlusive barriers with a window design demonstrated promising results for horizontal and vertical bone augmentation and keratinized gingiva formation, without the need for autologous bone grafts or primary wound closure.

## 1. Introduction

Tooth extraction triggers a series of biological processes that lead to progressive resorption of the alveolar bone, particularly within the first year [1] and continuing for up to five years [2]. This physiological bone remodeling can significantly compromise implant rehabilitation, especially when no early intervention is undertaken to preserve the volume of the post-extraction socket [3,4]. Clinical studies have demonstrated that unassisted healing often results in horizontal and vertical ridge deficiencies that hinder prosthetically driven implant placement and may necessitate additional regenerative procedures, thereby increasing treatment complexity, morbidity, and overall cost [5,6]. In this context, socket preservation and guided bone regeneration (GBR) techniques—such as distraction osteogenesis, block grafts, and GBR with resorbable or non-resorbable membranes—have proven to be key strategies for restoring adequate bone dimensions and enabling predictable implant rehabilitation [5,7].

Among the available reconstructive approaches, GBR has emerged as a widely adopted technique due to its favorable balance between clinical effectiveness and reduced donor-site morbidity when compared with autologous block grafting procedures [5,8,9]. The possibility of designing rigid customized titanium barriers tailored to the specific defect through CAD (Computer-Aided Design)/CAM (Computer-Aided Manufacturing) technologies has improved the stability of the regenerative space, promoting more controlled bone healing [10,11]. Systematic reviews have shown that titanium meshes allow predictable horizontal and vertical bone augmentation with high implant survival rates, although soft tissue dehiscence and barrier exposure remain technique-sensitive complications that require careful management [12,13].

From a biological standpoint, the therapeutic effectiveness of these devices is linked to their ability to maintain a secluded space where a stable blood clot can persist, angiogenesis can proceed, and soft-tissue collapse is minimized [7,14]. Rigid non-resorbable barriers, particularly those made of titanium, provide superior space-maintaining capacity compared with flexible membranes, especially in non–self-supporting and vertically demanding defects where mechanical stability is critical for successful regeneration [7,15].

From a biological standpoint, the therapeutic effectiveness of these devices is linked to their ability to maintain a secluded space where a stable blood clot can persist, angiogenesis can proceed, and soft tissue collapse is minimized [7,14]. Rigid non-resorbable barriers, particularly those made of titanium, provide superior space-maintaining capacity compared with flexible membranes, especially in non–self-supporting and vertically demanding defects where mechanical stability is critical for successful regeneration [7,15]. In this context, the synergy between the physical support provided by the titanium barrier and the biological properties of the stabilized blood clot creates a favorable microenvironment for angiogenesis and bone formation.

Within a biomimetic framework, GBR can be conceptualized as an engineered strategy that recreates core cues of physiological fracture healing and embryonic osteogenesis: (i) stabilization and protection of the blood clot as a provisional matrix; (ii) space maintenance to allow orderly cellular colonization; (iii) angiogenic support for nutrient and oxygen delivery; and (iv) selective cell exclusion to prevent competitive soft-tissue ingrowth [5,7,14]. These biological and mechanical requirements closely align with the PASS principles—primary wound closure, angiogenesis, space creation, and wound stability—which are essential for predictable bone regeneration and underscore the importance of incorporating clot stability, vascularization, and space maintenance into regenerative protocols [16].

Accordingly, the present protocol employs an autologous blood clot combined with β-tricalcium phosphate (β-TCP) as a biomimetic scaffold that stabilizes the blood clot—providing osteoconductive architecture, supporting neovascularization, and guiding osteoprogenitor migration—while the customized titanium barrier reproduces the mechanical boundary conditions (space protection and stability) required for early tissue organization [7,10,11,14]. β-TCP is a well-documented synthetic bone substitute characterized by biocompatibility, controlled resorption, and osteoconductive properties that support progressive bone replacement, making it particularly suitable for GBR applications in combination with autologous blood [17,18].

The use of biomaterials, whether of synthetic origin or derived from human or animal sources, also aims to reduce the collapse of newly formed bone tissue [6,19,20,21]. Biomaterials possess properties such as osteoconduction, osteoinduction, and osteogenesis [22]. One of the main characteristics of biomaterials such as tricalcium phosphate is osteoconduction, which, when combined with autologous blood, has been shown to enhance clot architecture and strengthen the regenerative environment [23]. Experimental and clinical evidence underscores that stabilization of the blood clot within a protected regenerative space is a critical determinant of angiogenesis, early bone formation, and overall GBR success [14,24].

These techniques are generally successful as long as the operator adheres to the PASS principles [16]. However, in complex alveolar defects, achieving tension-free primary closure may increase the risk of flap dehiscence; therefore, alternative strategies that prioritize space stability and controlled healing while limiting soft tissue manipulation have been proposed [7,25]. Moreover, the absence or insufficiency of keratinized mucosa has been associated with an increased risk of peri-implant complications. Recent meta-analyses indicate that lack of adequate keratinized mucosa is associated with increased plaque accumulation, greater soft tissue inflammation, mucosal recession, and a significantly higher risk of peri-implantitis, emphasizing the importance of soft tissue evaluation and management in GBR-based implant rehabilitation [26,27].

The development of digital workflows has represented a major advance in GBR by enabling defect-specific planning, reducing surgical time, and improving clinical predictability [15,28,29,30]. Comparative evidence suggests that customized CAD/CAM titanium meshes provide bone gains comparable to conventional meshes while showing a tendency toward reduced complication rates and improved fit, supporting their adoption in digitally driven regenerative protocols [12]. In this protocol, the window-modified occlusive barriers allow direct clinical monitoring and targeted biological interventions—standardized irrigation and controlled rebleeding to renew the clot—aimed at clot stability, support of neovascularization, and preservation of the regenerative space. These intended effects are linked to measurable outcomes, namely horizontal and vertical bone gain quantified on CBCT and histological evidence of vascularized newly formed bone [11,14].

In this context, the present study was designed as a prospective case series with the primary objective of quantifying horizontal and vertical bone gain in complex alveolar ridge defects treated using a guided bone regeneration protocol based on customized titanium occlusive barriers with a window design, combined with the autologous blood clot and β-tricalcium phosphate, through superimposition of preoperative and postoperative CBCT scans. As a secondary objective, the clinical formation and maintenance of keratinized gingiva at the regenerated sites were evaluated. The working hypothesis was that this protocol is feasible and allows the achievement of clinically relevant bone gain with a low rate of major complications, even in the absence of autologous bone grafts and primary wound closure.

## 2. Materials and Methods

### 2.1. Study Design and Participants

This investigation was designed as a prospective case series and was carried out between October 2022 and August 2023 in accordance with the STROBE (acronym for Strengthening the reporting of observational studies in epidemiology) recommendations (Supplementary Material: Document S1). A total of 13 patients were enrolled, presenting alveolar bone defects classified as Terheyden type 2/4 or 3/4 [31], a classification system that categorizes defects according to the number of residual bony walls. Specifically, type 2/4 defects are characterized by the presence of two remaining walls, whereas type 3/4 defects correspond to sites with only one residual wall, reflecting moderate to severe three-dimensional alveolar deficiencies.

These defect configurations were intentionally selected because they represent clinical scenarios in which predictable space maintenance and adequate mechanical stability are critical, thereby constituting appropriate indications for evaluation using an occlusive titanium barrier. Overall, 14 surgical interventions were performed to manage 28 alveolar sites, as one patient underwent treatment with two separate and independent barriers.

All participants followed the predefined clinical schedule without deviations. Barrier removal was performed at six months postoperatively, radiographic evaluation using CBCT was conducted at eight months, and implant placement together with bone biopsy retrieval took place at ten months.

Eligibility criteria included an age of 18 years or older, periodontal stability at the time of inclusion, and the patient’s commitment to comply with the established follow-up protocol.

Exclusion criteria included pregnancy, moderate-to-heavy smoking, active periodontitis, use of medications known to impair healing, local infection, mobility of adjacent teeth, or inability to attend follow-up visits. Patients with systemic conditions known to compromise wound healing, such as uncontrolled diabetes mellitus or uncontrolled hypertension, were excluded from the study; accordingly, none of the enrolled patients presented with these conditions. Light smokers (<10 cigarettes per day) were not excluded; however, no active smokers were included in the present cohort. All 13 recruited patients (28 sites) successfully completed the study protocol and were included in the final analysis, with no missing data nor post-enrollment exclusions.

The study protocol was approved by the Research Ethics Committee of Jaén (1794-N-22, 21 September 2022), and all participants provided written informed consent for both the surgical procedure and study participation.

**Figure 1:**
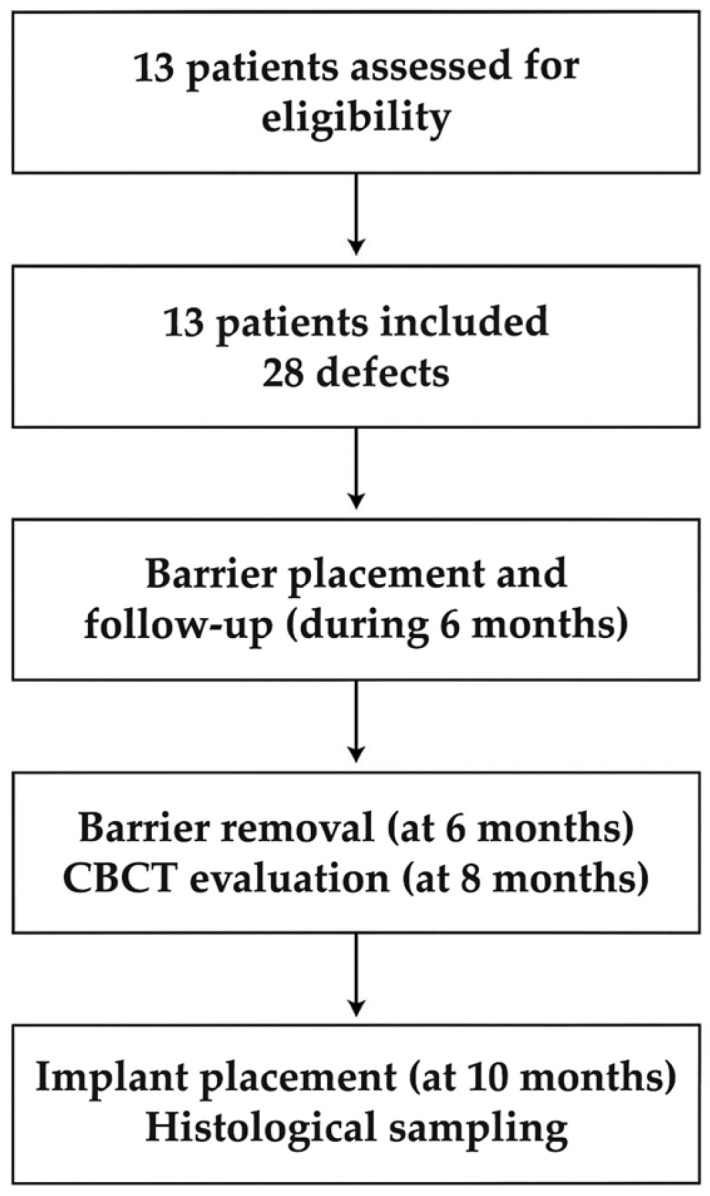
Participant flow diagram according to the STROBE statement, summarizing patient inclusion, treatment allocation, follow-up, and data analysis.

The study flow is summarized in Figure 1.

### 2.2. Outcomes

The primary outcomes were horizontal and vertical bone gain, assessed radiographically between the preoperative baseline (T0) and the final follow-up (T1), at a mean of 8 months. Secondary outcomes included the presence of keratinized mucosa (at 10 months), postoperative complications (e.g., local infection), and the clinical stability of the barrier.

### 2.3. Barrier Design and Fabrication

**Figure 2:**
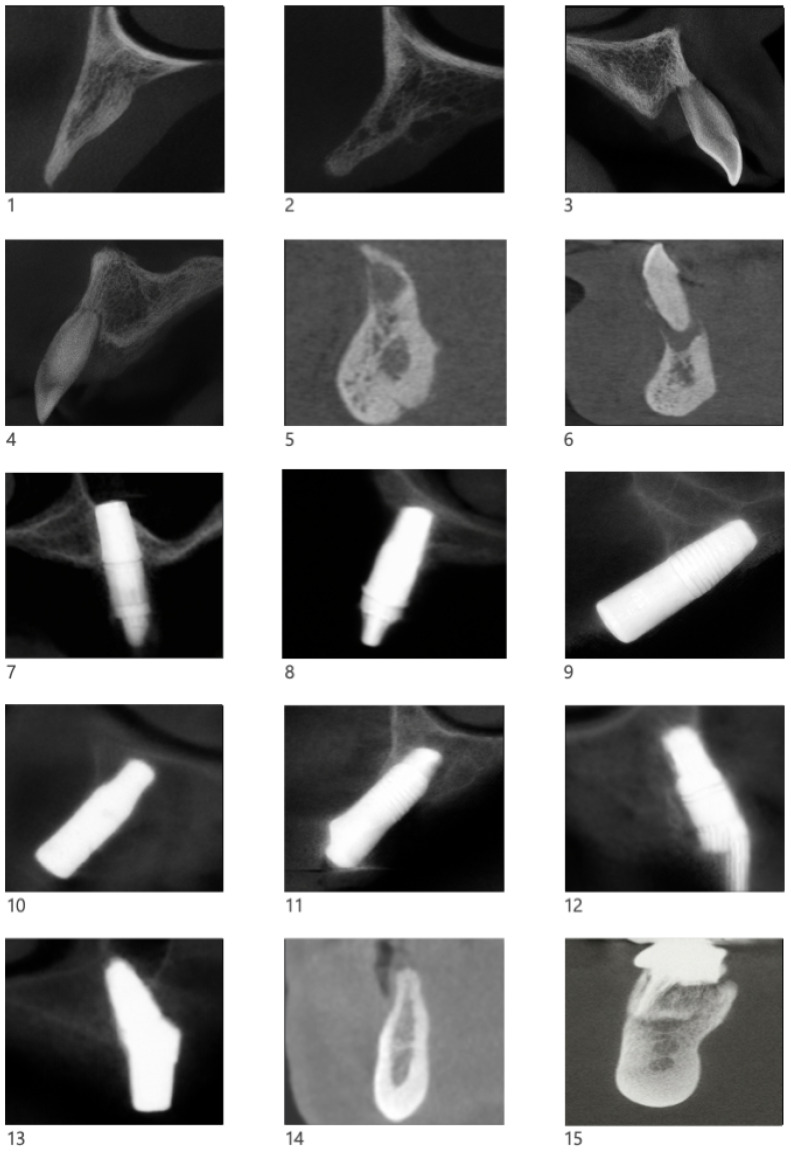

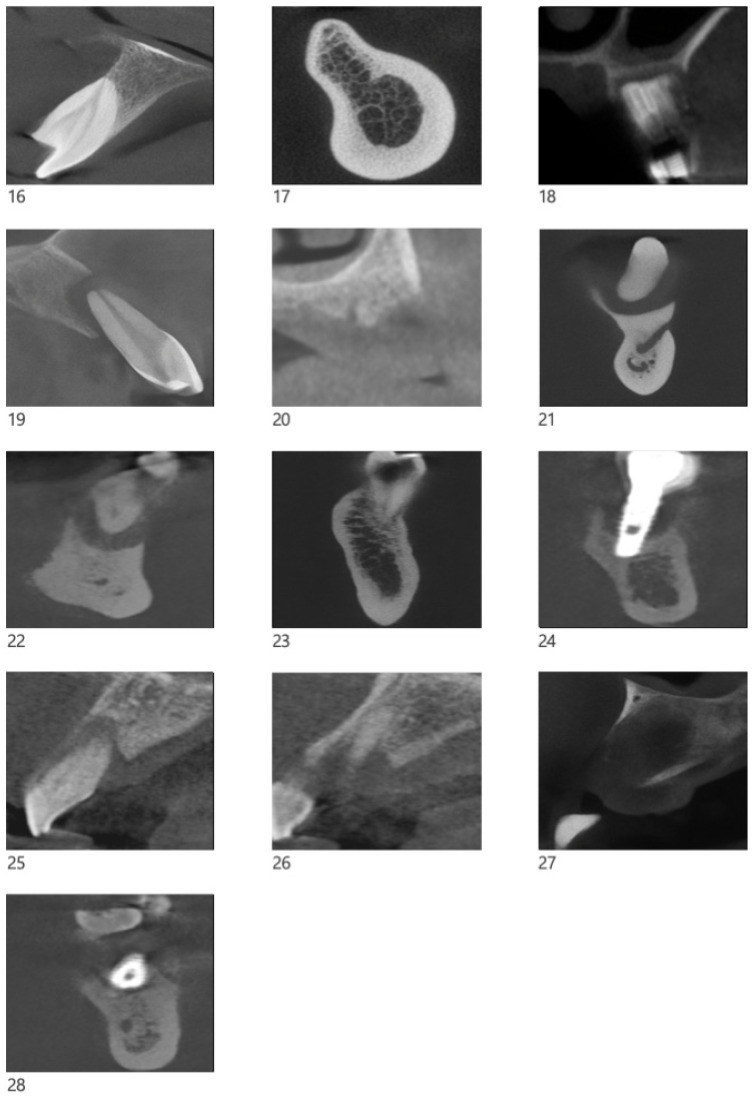
Preoperative sagittal CBCT images showing the residual atrophic area prior to surgery. Numbers 1 to 28 correspond to the 28 cases described, grouped into 13 patients. Using the dental numbering system for identification, the location of each case is as follows: Case 1: Tooth 13; Case 2: Tooth 15; Case 3: Tooth 12; Case 4: Tooth 22; Case 5: Tooth 32; Case 6: Tooth 42; Case 7: Tooth 16; Case 8: Tooth 15; Case 9: Tooth 11; Case 10: Tooth 22; Case 11: Tooth 23; Case 12: Tooth 24; Case 13: Tooth 26; Case 14: Tooth 44; Case 15: Tooth 46; Case 16: Tooth 21; Case 17: Tooth 36; Case 18: Tooth 21; Case 19: Tooth 24; Case 20: Tooth 16; Case 21: Tooth 33; Case 22: Tooth 35; Case 23: Tooth 35; Case 24: Tooth 36; Case 25: Tooth 11; Case 26: Tooth 12; Case 27: Tooth 22; Case 28: Tooth 36.

Preoperative assessment included medical history, intraoral photographs, intraoral scanning (PRISMECAN), and low-dose cone-beam computed tomography (CBCT) (Figure 2).

CBCT DICOM files were processed using Simpleware ScanIP (Synopsys, Sunnyvale, USA; v7.0) to generate a 3D mask, which was exported in STL format. The STL file was imported into Rhinoceros 3D (Asuni Soft S.L., Barcelona, Spain; v6.0) for the digital design of the customized Osteophoenix titanium barrier.

**Figure 3:**
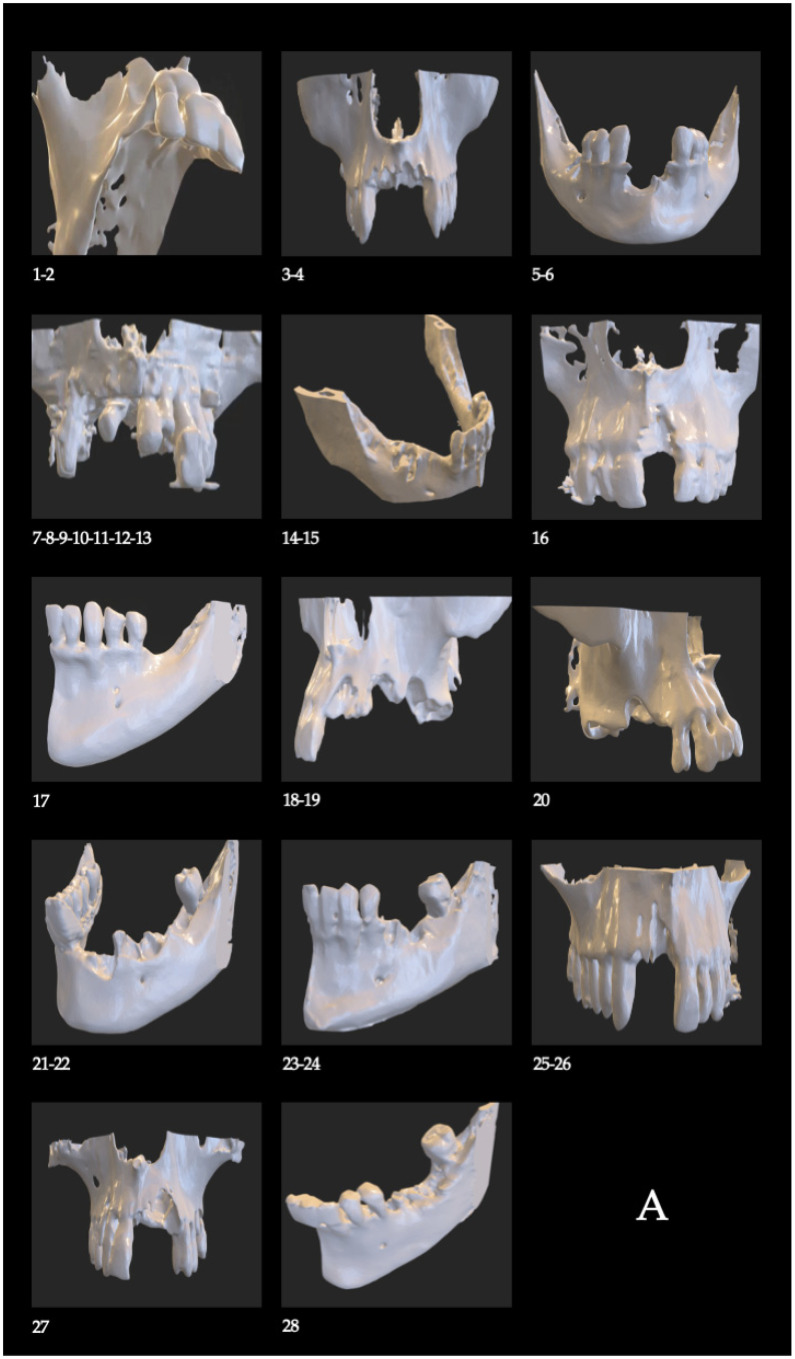

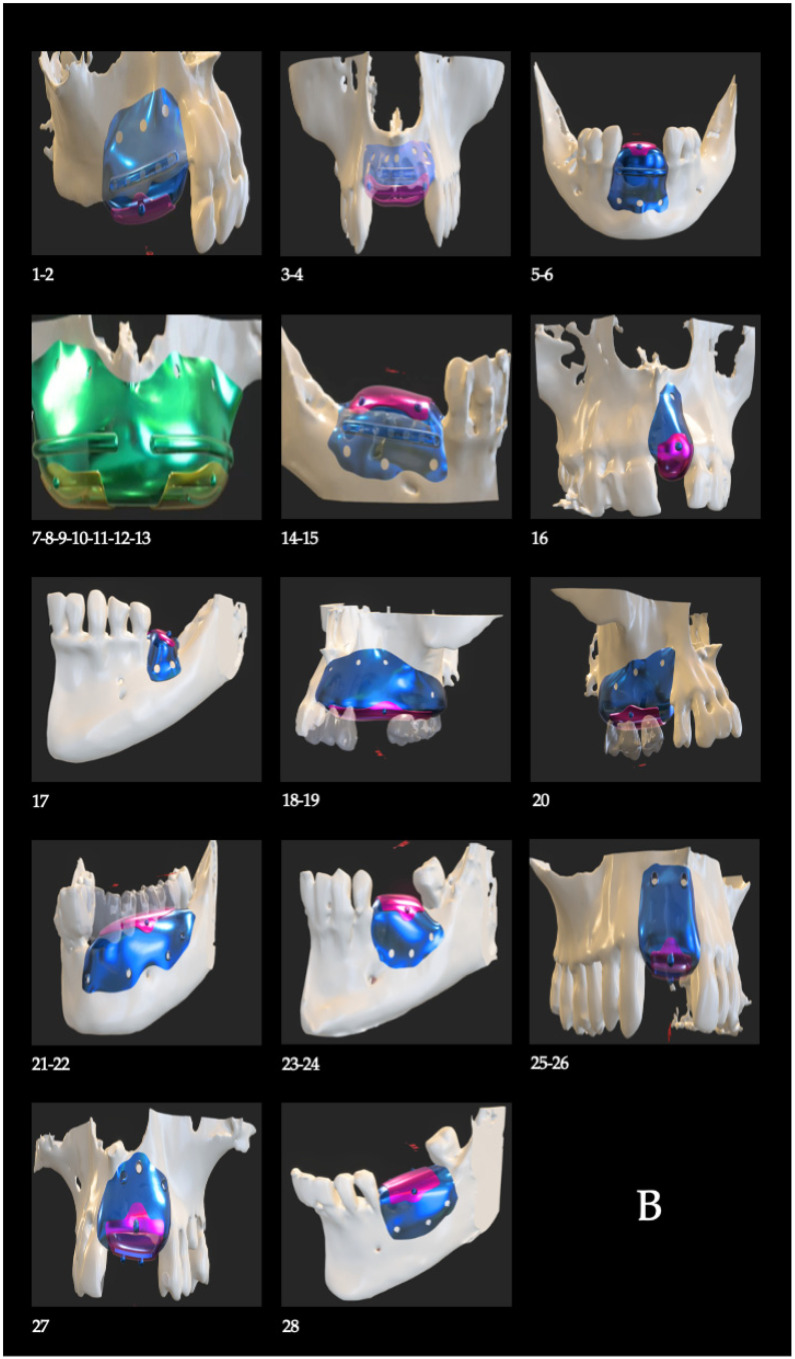
(**A**) Virtual design of bone defects and fabrication of biomodels using stereolithography. (**B**) Virtual design of the customized titanium barriers adapted to the defects. The design illustrates the customized fit and the integrated window. Numbers 1 to 28 correspond to the 28 cases described, grouped into 13 patients. Using the dental numbering system for identification, the location of each case is as follows: Case 1: Tooth 13; Case 2: Tooth 15; Case 3: Tooth 12; Case 4: Tooth 22; Case 5: Tooth 32; Case 6: Tooth 42; Case 7: Tooth 16; Case 8: Tooth 15; Case 9: Tooth 11; Case 10: Tooth 22; Case 11: Tooth 23; Case 12: Tooth 24; Case 13: Tooth 26; Case 14: Tooth 44; Case 15: Tooth 46; Case 16: Tooth 21; Case 17: Tooth 36; Case 18: Tooth 21; Case 19: Tooth 24; Case 20: Tooth 16; Case 21: Tooth 33; Case 22: Tooth 35; Case 23: Tooth 35; Case 24: Tooth 36; Case 25: Tooth 11; Case 26: Tooth 12; Case 27: Tooth 22; Case 28: Tooth 36.

The barrier was manufactured from a Ti6Al4V alloy via laser sintering (~350 °C, porosity 30–60 μm). The final device had a thickness of 0.7 mm, with customized length and window dimensions. Post-processing included pink anodization, milling (CAM Magics 23, Materialise NV, Leuven, Belgium), sintering at 1450 °C, and final sterilization in an autoclave (Tinhero 16 Class B, Runyes Medical Instrument Co., Ltd., Ningbo, China) at 134 °C (Figure 3).

A simplified schematic representation of the customized titanium occlusive barrier with window design is provided in Supplementary Figure S1 for enhanced methodological clarity.

### 2.4. Surgical Protocol

#### 2.4.1. Barrier Placement Surgery

**Figure 4:**
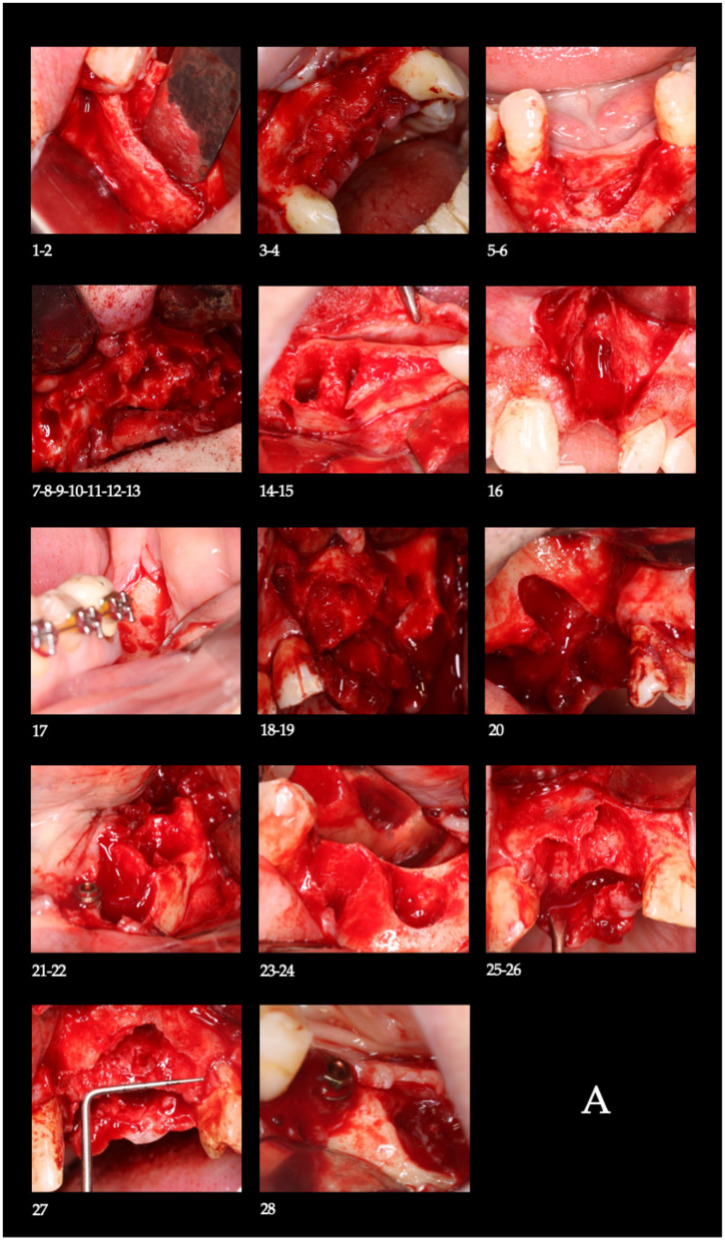

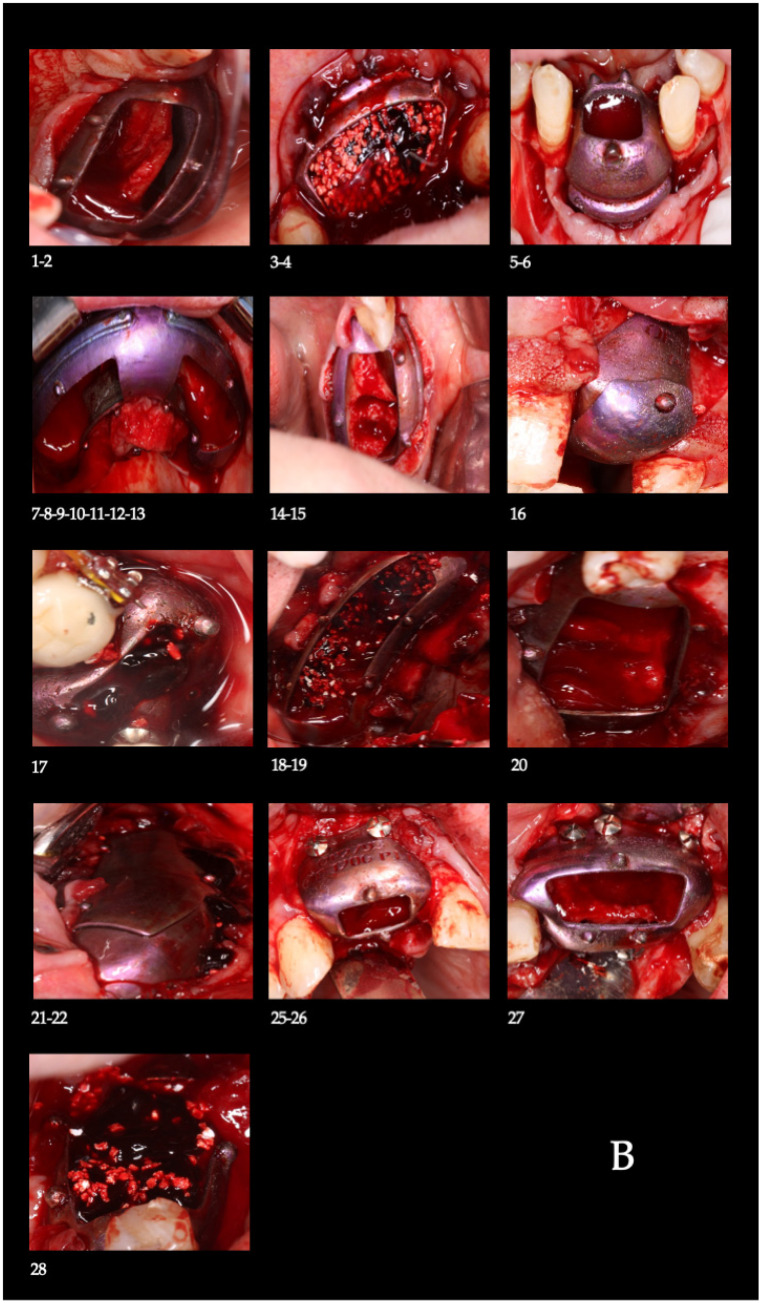

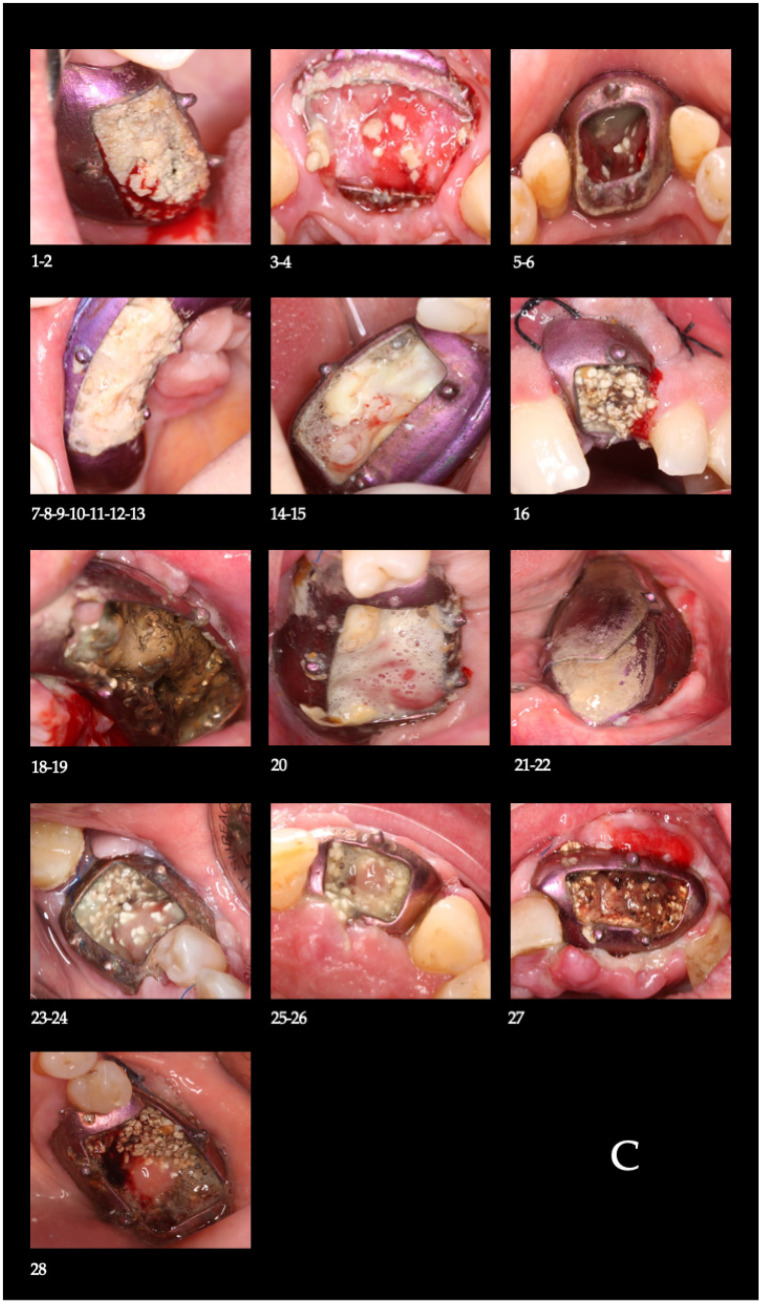

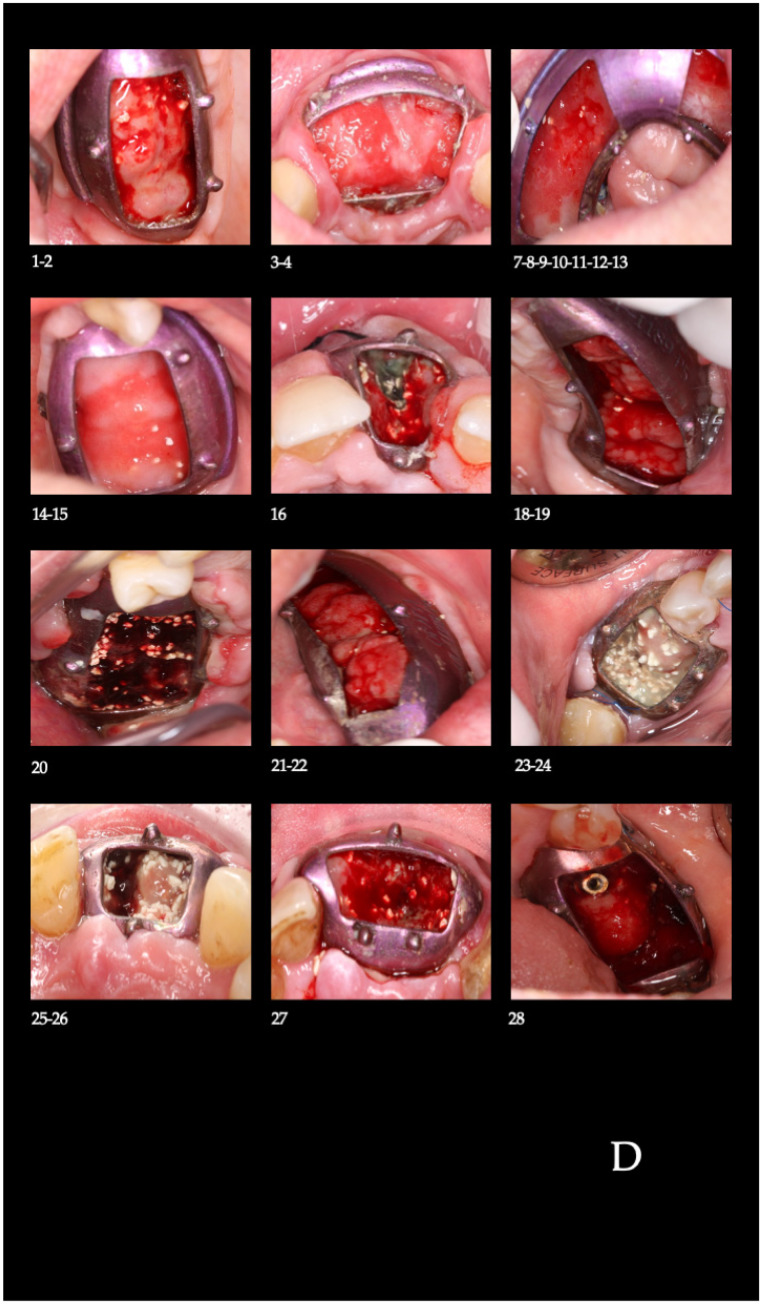

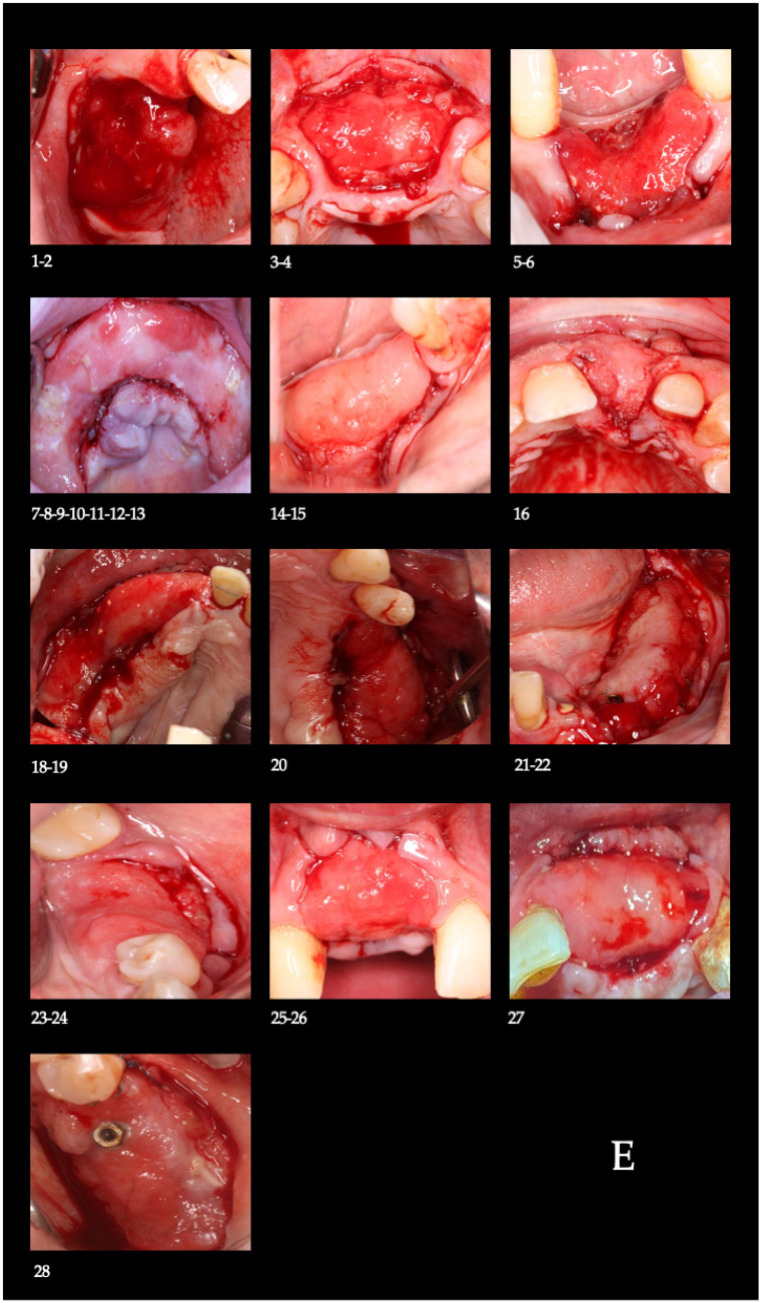

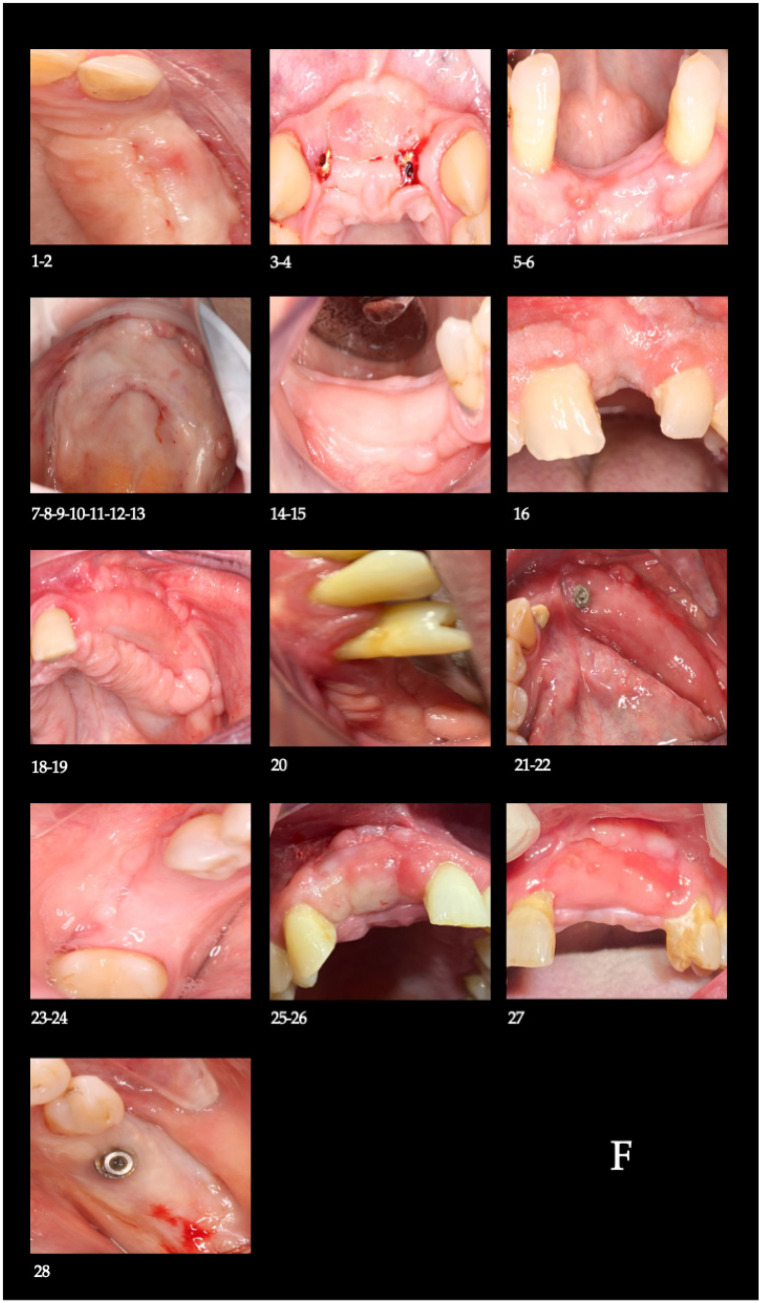
(**A**) Bone defect in the area to be regenerated. (**B**) Placement of the customized titanium barriers in the guided bone regeneration site. (**C**) Clinical view of the barrier window during the follow-up protocol. (**D**) Irrigation with sterile saline solution prior to deepithelialization and filling with tricalcium phosphate. (**E**) Removal of the barrier after the consolidation period. (**F**) Placement of the implants in the regenerated site. Numbers 1 to 28 correspond to the 28 cases described, grouped into 13 patients.

Following local anesthesia (4% articaine), a full-thickness mucoperiosteal flap was elevated. Bone decortication was performed to promote angiogenesis [32]. The customized barrier was fixed with titanium screws (1.5 mm diameter) using a 1.3 mm drill under saline irrigation. A graft mixture of autologous blood clot and tricalcium phosphate was packed into the defect, the barrier window was closed, the flap was sutured with interrupted non-resorbable 4-0 simple stitches, and additional approximation sutures were placed along the crestal incision (Figure 4A,B).

#### 2.4.2. Postoperative Care and Follow-up Timeline

Following surgery, patients received amoxicillin-clavulanic acid (875/125 mg every 12 h) and analgesics for one week. No protocol deviations regarding the surgical procedure, postoperative medication, or follow-up schedule occurred in any of the included cases. A specific follow-up protocol was implemented.

- Day 7: Irrigation with sterile saline solution (SSS) and removal of loose debris.
- Day 16: Loss of osteoid volume and necrotic blood was observed (Figure 4C). Irrigation with SSS was performed (Figure 4D), followed by a protocolized deepithelialization of the superficial epithelial layer through the barrier window. This procedure was performed without local anesthesia to induce bleeding and subsequent clot formation, a step intended to prevent epithelial migration into the regenerative compartment. Once blood coagulation had occurred, tricalcium phosphate was reapplied. This protocol was designed to prevent epithelial migration into the regenerative compartment, a known inhibitor of osteogenesis [24], while the β-TCP reapplication served to maintain the osteoconductive scaffold and support fresh clot formation during critical healing phases. Operationally, these steps are the biomimetic levers of the protocol (clot renewal and soft-tissue exclusion within a space-stable compartment) and were predefined to favor angiogenesis and osteoprogenitor colonization; their effect was later evaluated through CBCT-based dimensional gains and histology.
- Days 25, 33, 42, 50, and 59: The irrigation and deepithelialization protocol was repeated.
- From month 3 to 6: Visits were scheduled every 21 days for irrigation only. After six months, the barrier was sectioned into two segments for extraction under local anesthesia (Figure 4E).

All surgeries and follow-up interventions, including the protocolized deepithelialization steps, were performed by the same lead surgeon to maintain procedural consistency.

#### 2.4.3. Final Evaluation and Implant Placement

In strict adherence to the protocol, a second CBCT scan (T1) was acquired for radiographic evaluation at eight months after the initial surgery. Implant placement and bone biopsy were performed two months after barrier removal (ten months post-initial surgery) (Figure 4F). Surgical guides were used to protect the regenerated site. This timeline was specifically designed to evaluate the regenerative outcomes of the technique, with the follow-up period focused on bone formation prior to functional loading. Implant placement was performed only when the regenerated ridge allowed prosthetically driven implant insertion with adequate primary stability, as assessed clinically and radiographically at the time of surgery. Although no single numeric cutoff was predefined, this corresponded in practical terms to achieving a minimum horizontal ridge width sufficient to accommodate a standard-diameter implant (approximately ≥6 mm) and a vertical bone height allowing placement of implants of at least 8–10 mm without encroachment on adjacent anatomical structures. All treated sites met these clinical criteria and successfully underwent implant placement according to the predefined protocol.

### 2.5. Definitions and Ascertainment of Adverse Events

Adverse events were systematically defined and ascertained through clinical examination at every follow-up visit (Days 7, 16, 25, 33, 42, 50, 59, and then monthly until barrier removal) by the lead surgeon. The specific definitions were as follows:

- Screw Loosening: Clinical mobility of the fixation screw upon palpation with a dental probe, requiring intervention.
- Window Detachment: Complete separation of the barrier’s window from the main body of the device, observed clinically.
- Infection: Presence of purulent discharge, accompanied by erythema, swelling, and pain at the surgical site.
- Exposure: Full-thickness loss of mucosal coverage resulting in complete visibility of the barrier membrane.
- Partial Exposure: Incomplete loss of mucosal coverage where the barrier becomes visible but is not fully exposed due to a small mucosal breach.
- Dehiscence: Rupture of the surgical suture line leading to wound separation, without direct exposure of the underlying barrier.
- Barrier Removal: The planned surgical procedure to explant the device after the consolidation period. Unplanned early removal was defined as the necessity to remove the barrier prior to the scheduled 6-month time point due to complications such as infection, exposure, or loss of stability that could not be resolved.

### 2.6. Data Acquisition and Measurement Methods

#### 2.6.1. Radiographic Analysis

Volumetric and linear measurements of bone gain were obtained from the T0 and T1 CBCT scans. All scans were acquired using a low-dose protocol with a standardized voxel size of 0.25 mm and a field of view of 8 cm × 8 cm to ensure high resolution and minimize radiation exposure. Tomographic data were exported in DICOM format. These DICOM files were analyzed using the medical software 3DSlicer, version 5.2.1 (https://slicer.org, accessed on 7 February 2024) [33]. For the specific quantification of the grafted volume, the following digital workflow was implemented:

- Segmentation: The pre-operative (T0) and post-operative (T1) scans were independently segmented using the software’s thresholding tool. A consistent Hounsfield Unit (HU) threshold range of 250–350 HU was applied across all scans to generate distinct three-dimensional models of the osseous anatomy at each time point. To mitigate metal artifacts from the titanium screws and barrier, the scans were processed using the software’s built-in metal artifact reduction algorithm prior to segmentation, and manual refinement was performed slice-by-slice to exclude any residual artifact-induced noise.
- Registration: The resulting T0 and T1 models were precisely co-registered using an anatomy-based rigid registration method. The iterative closest point (ICP) algorithm was employed, aligning the models based on the stable native bone structures adjacent to the defect site, specifically the cortical bone borders and intact anatomical landmarks such as the mandibular canal or sinus floor when available. The accuracy of this co-registration was quantified by calculating the Target Registration Error (TRE), which yielded a mean value of 0.15 mm (±0.08 mm).
- Volumetric Calculation: The volumetric difference between the registered T1 and T0 segments was calculated using a Boolean subtraction operation. The resulting output, representing the graft volume, was resegmented to isolate it definitively and was exported in STL format for both qualitative visualization and precise quantitative analysis [34].
- Blinding: To minimize bias, the operators performing the segmentations and measurements were blinded to the time point (T0 or T1) of the anonymized and randomized scans.

#### 2.6.2. Reliability Analysis

To assess the reliability of the volumetric measurements, an analysis of intra- and inter-observer agreement was conducted. A random subset of 10 scans (approximately 35% of the total sites) was selected. A single blinded operator repeated the entire segmentation and measurement process for this subset after a two-week washout period to determine intra-observer reliability. A second, independent blinded operator performed the same procedures on the subset to determine inter-observer reliability. The reliability of the resulting volumetric measurements was quantified using a two-way mixed-effects model for absolute agreement Intraclass Correlation Coefficient (ICC). The analysis demonstrated excellent reliability, with an intra-observer ICC of 0.985 (95% CI: 0.963–0.995) and an inter-observer ICC of 0.972 (95% CI: 0.928–0.990).

#### 2.6.3. Histological Analysis

For the qualitative histological evaluation of bone formation, core biopsies were procured during the implant placement surgery, which was performed four months following barrier removal. A total of 14 biopsies were planned, corresponding to the 14 regenerated sites; however, in one case the biopsy specimen could not be retrieved from the trephine and was therefore unavailable for analysis. Consequently, histological evaluation was performed on 13 biopsy specimens. Using a trephine bur, drilling was conducted to the planned implant depth to harvest the bone sample. The obtained specimens were fixed in a 10% neutral buffered formalin solution to preserve tissue architecture. Subsequently, the samples were dehydrated, embedded in paraffin blocks, and sectioned into 5 µm-thick slices. These sections were then stained with hematoxylin–eosin to enable a qualitative assessment of the newly formed bone matrix, osteocyte lacunae, and overall tissue morphology. In cases where multiple biopsies were available from a single patient, a single representative specimen exhibiting optimal technical quality and preservation was selected for analysis to prevent statistical redundancy, as samples from the same individual share an identical biological milieu.

### 2.7. Statistical Analysis

Statistical analyses were conducted using R software (version 4.4.2; R Core Team, 2020), freely available at https://www.r-project.org/ (accessed on 5 May 2024). Data normality was assessed using the Shapiro–Wilk test. This investigation was conceived as an exploratory prospective case series and was not powered to detect specific effect sizes; accordingly, no a priori sample size (power) calculation was performed. Primary analyses employed linear mixed-effects models to account for the hierarchical data structure (28 defects nested within 14 barriers nested within 13 patients), including random intercepts at the patient and barrier levels. Given the small number of clusters (*n* = 13 patients), there is a risk of model overfitting and unstable variance estimates; therefore, model complexity was intentionally limited and all *p* values and confidence intervals should be interpreted with caution within this exploratory, hypothesis-generating framework. As a sensitivity analysis to confirm robustness, patient-level aggregated data were analyzed using the Wilcoxon signed-rank test for paired samples. Effect sizes were estimated using the Hodges–Lehmann estimator for the median difference, with 95% confidence intervals obtained via percentile bootstrap (5000 replicates). For reliability assessment of CBCT measurements, intra- and inter-observer agreement was evaluated using a two-way mixed-effects model for absolute-agreement intraclass correlation coefficient (ICC). Interpretation of ICC values followed established guidelines: <0.50 = poor, 0.50–0.75 = moderate, 0.75–0.90 = good, and >0.90 = excellent reliability. The standard error of measurement (SEM) was calculated as SEM = SD × √(1 − ICC), where SD represents the standard deviation of the measurements. Complication rates (reported as proportions) are presented with 95% confidence intervals calculated using the Wilson score interval method. To explore potential confounding factors, secondary analyses were performed using Kruskal–Wallis tests to assess the influence of defect classification (Terheyden type 2/4 vs. 3/4) and anatomical location (anterior vs. posterior) on the primary outcomes of horizontal and vertical bone gain. Data are expressed as mean ± standard deviation or as frequencies/percentages. Absolute bone gain was calculated as the postoperative minus preoperative difference (mm). Statistical significance was set at *p* < 0.05 for all analyses.

### 2.8. Literature Search Strategy for Contextualization

A PRISMA-S-inspired description of the literature search conducted to contextualize the study findings has been provided in the Supplementary Material (Document S2).

## 3. Results

The mean age of the 13 patients (63.63% men) included in the study was 50.16 years, with a minimum age of 27 and a maximum age of 73 years.

### 3.1. Radiological Observation

**Figure 5:**
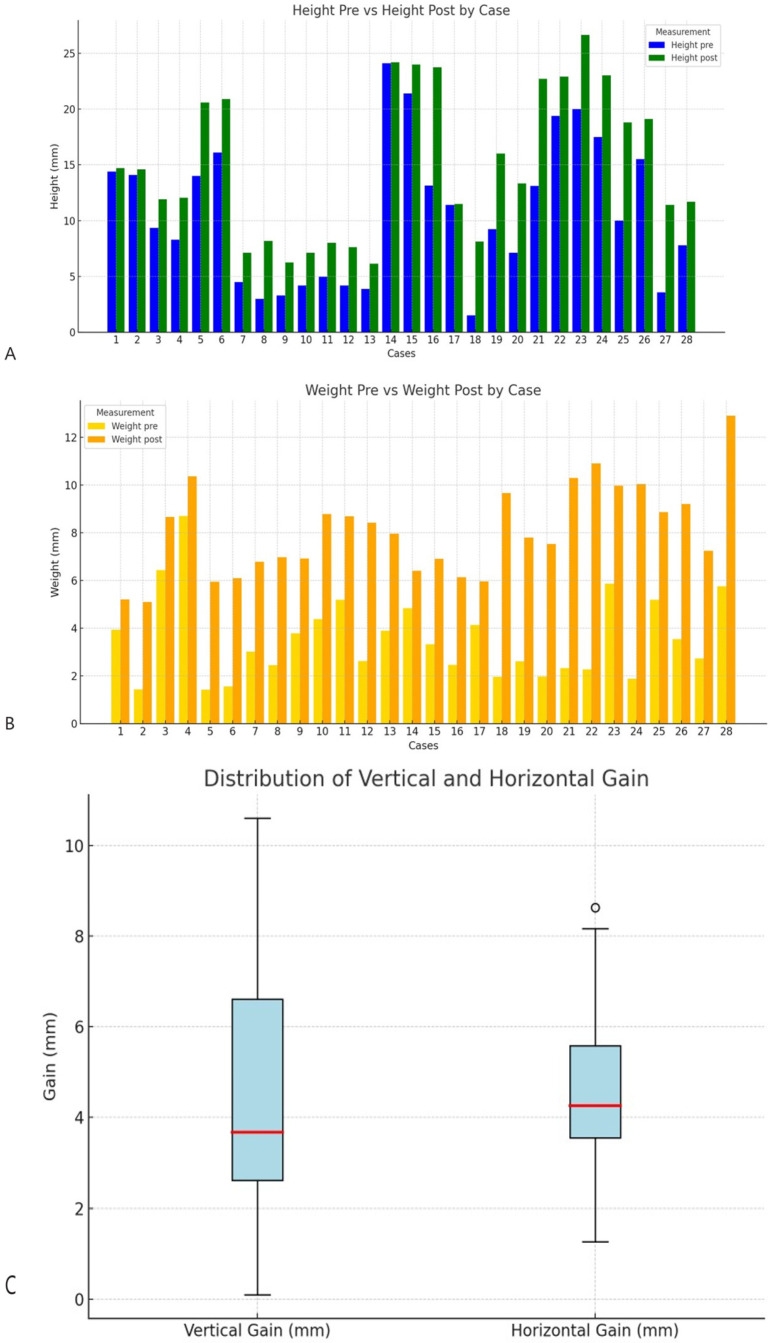
(**A**) Pre- and postoperative vertical bone dimensions (height) for each individual case. Bars represent the mean vertical measurement before (blue) and after (green) the procedure. (**B**) Pre- and postoperative horizontal bone dimensions (width) for each individual case. Bars represent the mean horizontal measurement before (yellow) and after (orange) the procedure. (**C**) Boxplots of vertical and horizontal bone gain. The boxes represent the IQR, the horizontal line inside the box marks the median, and the whiskers extend to the minimum and maximum values.

The procedure yielded a statistically significant increase in bone dimensions. The horizontal width (Figure 5A) increased from a preoperative mean of 3.56 ± 1.38 mm (median: 3.17 mm; range: 1.42–8.70 mm) to 8.06 ± 1.96 mm (median: 7.88 mm; range: 5.10–12.90 mm) postoperatively, resulting in a mean gain of 4.50 ± 2.02 mm (range: 1.27–8.63 mm; IQR: 2.03 mm). Similarly, the vertical height (Figure 5B) increased from 10.68 ± 6.17 mm (median: 9.69 mm; range: 1.50–24.10 mm) to 15.09 ± 5.89 mm (median: 13.97 mm; range: 6.15–26.63 mm), with a mean gain of 4.40 ± 2.82 mm (range: 0.10–10.60 mm; IQR: 3.99 mm). The Hodges-Lehmann estimator, which reflects the median gain, was 4.60 mm (95% CI [2.57, 6.14]) for the horizontal dimension and 4.66 mm (95% CI [2.14, 6.55]) for the vertical dimension, confirming that these gains were highly statistically significant (Wilcoxon signed-rank test, *p* = 0.00097 for both). From a clinical perspective, these postoperative dimensions were sufficient to allow prosthetically driven implant placement at all sites, confirming that the regenerated ridges reached clinically meaningful thresholds for implant rehabilitation in moderate to severe defects.

Linear mixed-effects models accounting for the clustered data structure showed significant bone gain for both horizontal (estimate: 4.52 mm, 95% CI: 3.12–5.92, *p* < 0.001) and vertical dimensions (estimate: 4.38 mm, 95% CI: 2.89–5.87, *p* < 0.001).

In the individual case representation (Figure 5A,B), a consistent increase in postoperative measurements was observed in both height and width compared with preoperative values, reinforcing the overall trend of gain after the procedure.

In the boxplots (Figure 5C), a more dispersed distribution was observed in vertical gain, with maximum values exceeding 10 mm, while horizontal gain showed less variability, although with one case reaching values close to 8 mm.

The complete descriptive values of the pre- and postoperative measurements, as well as the horizontal and vertical gains, are presented in Table 1.

### 3.2. Histological Observation

**Figure 6:**
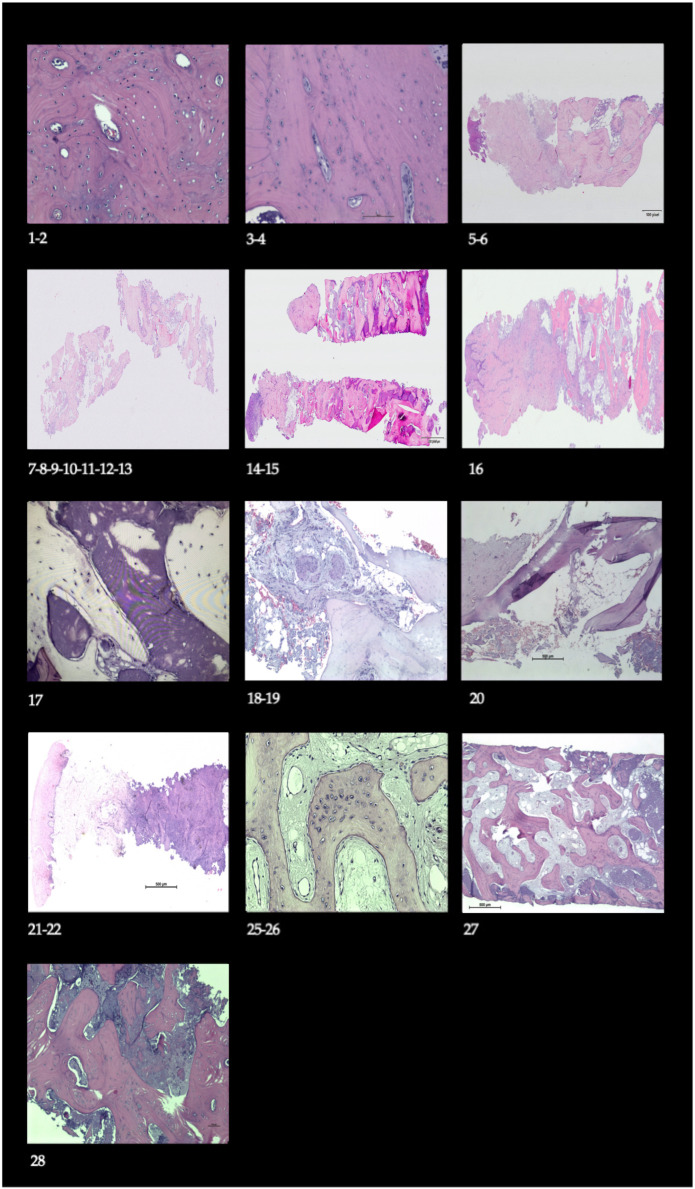
Histological findings from representative cases. The biopsies revealed newly formed bone with thick trabeculae (Case 7). In some samples, persistence of non-resorbed inorganic material was noted within intertrabecular spaces (Case 17), together with osteocytes in lacunae and active osteoblasts on the surface (Case 25), as well as keratinized gingiva (Cases 5, 16, and 21).

Histological examination of the biopsies (Figure 6) revealed the presence of newly formed bone with thick trabeculae. In some samples, remnants of non-resorbed inorganic material were observed within the intertrabecular spaces. Osteocytes within lacunae and active osteoblasts on the surface were identified, together with keratinized gingiva. Importantly, no inflammatory reaction or fibrous encapsulation was detected, indicating proper tissue integration after guided bone regeneration.

### 3.3. Complications and Safety Outcomes

Complications were ascertained according to the predefined definitions. One case of screw loosening (Case 17) and one case of window detachment (Case 3) were recorded. Both were successfully managed by screw replacement and window repositioning, respectively, with no subsequent compromise to the final outcome. No cases of infection, exposure, partial exposure, dehiscence, or unplanned barrier removal were observed during the entire follow-up period.

The complication rates were calculated as follows:

- Per-patient analysis (*n* = 13 patients): The overall complication rate was 15.4% (2 out of 13 patients; 95% CI [2.7%, 46.4%]).
- Per-site analysis (*n* = 28 sites): The overall complication rate was 7.1% (2 out of 28 sites; 95% CI [1.3%, 23.5%]). The specific rates were 3.6% (95% CI [0.2%, 19.0%]) for screw loosening and 3.6% (95% CI [0.2%, 19.0%]) for window detachment.

## 4. Discussion

The use of β-TCP in this study is based on its osteoconductive architecture, which supports clot stability, cell migration, and angiogenesis, while undergoing controlled resorption that balances volume maintenance with replacement by vital bone [14,17,18]. In our protocol, β-TCP functioned as the scaffold component within a space-stable compartment provided by the customized titanium barrier.

Many conventional GBR techniques rely on autologous bone harvesting, either alone or in combination with biomaterials, which increases surgical complexity, operative time, and patient morbidity [5,8,9]. In contrast, the present protocol achieved bone regeneration without the use of autologous bone grafts, relying instead on an autologous blood clot and β-TCP under a rigid occlusive barrier. This approach simplifies the surgical workflow while preserving the fundamental requirement of space stability, which is widely recognized as a critical determinant of GBR success [5,7].

Soft-tissue management remains a key challenge in conventional GBR because primary closure frequently requires advanced flap release and periosteal scoring to achieve tension-free adaptation, with potential effects on surgical complexity and morbidity [16]. In contrast, our window-based protocol deliberately avoided primary closure and enabled scheduled, aseptic chairside access to the regenerative compartment, which facilitated two predefined, mechanism-oriented actions: (i) controlled deepithelialization to limit epithelial down-growth into the defect space, and (ii) renewal of the blood clot together with maintenance of an osteoconductive scaffold through β-TCP reapplication, thereby supporting early angiogenesis and osteoprogenitor migration within a space-stable compartment created by the customized titanium barrier [17,24]. This open-window strategy has been described previously for occlusive barriers as a way to simplify flap management while enabling direct monitoring of healing [11,25]. In our cohort, despite the theoretical risk of contamination associated with repeated access, no infections and no unplanned barrier removals occurred during follow-up; only two minor hardware-related events (one screw loosening and one window detachment) were observed and resolved without impact on the final outcome, a safety profile consistent with the CBCT-measured dimensional gains and qualitative histological findings reported in this study. Overall, these observations support the operational link between the protocol’s biomimetic levers (space maintenance and clot stabilization with soft-tissue control) and the measurable outcomes of horizontal/vertical bone gain and tissue integration [5,7].

In this case series, mean horizontal and vertical gains were 4.50 mm and 4.40 mm, respectively, and keratinized gingiva formed at all sites. These values are slightly lower than those reported in our previous cohort using the same technique; this difference may reflect baseline defect morphology, patient selection, or follow-up timing rather than a protocol change [11]. Even so, the current outcomes confirm feasibility and reproducibility in a different patient set under routine conditions.

From a clinical standpoint, the magnitude of augmentation observed in this cohort aligns with dimensions typically targeted for standard implant therapy. Common implant diameters (≈3.5–4.5 mm) within widely adopted GBR and augmentation workflows require achieving a post-augmentation ridge width compatible with prosthetically driven placement and circumferential bone support, as emphasized across vertical augmentation principles, membrane/mesh-based GBR overviews, customized CAD/CAM mesh series, and block-graft protocols [5,7,15]. In our study, the mean postoperative ridge width was 8.06 ± 1.96 mm with a mean horizontal gain of 4.50 ± 2.02 mm, and the mean postoperative ridge height was 15.09 ± 5.89 mm with a mean vertical gain of 4.40 ± 2.82 mm; these dimensions were sufficient to permit prosthetically guided implant placement at all treated sites according to protocol, underscoring the clinical relevance of the achieved regeneration in moderate-to-severe defects.

Reported outcomes for established augmentation protocols vary depending on technique and operator experience. Block-graft and vertical augmentation protocols typically achieve substantial horizontal and vertical gains but require autologous bone and primary closure [8,9]. Customized mesh approaches improve space maintenance but may show exposure rates that depend on flap management [15]. In our cohort, the window-based barrier achieved clinically relevant dimensional gains with a low complication rate and no infections, at the cost of additional chairside follow-up inherent to the open-window design. Direct effectiveness comparisons are not possible due to the case-series design and cohort differences [5,7].

To contextualize the outcomes of the present cohort, Table 2 provides a comparative summary of horizontal and vertical bone gains, complication profiles, and key procedural characteristics reported for conventional GBR techniques versus the customized titanium occlusive barrier approach [5,7,8,9,10,15,28,31].

The radiographic evaluation used in this study also presents certain limitations, as it may not differentiate between mature and immature bone and may be affected by the presence of materials or devices that interfere with image interpretation. Therefore, complementing radiographic assessment with histological analysis is essential, as it provides a more precise and comprehensive understanding of the bone regeneration process. This constitutes one of the strengths of the present study.

The histological evaluation substantiates the successful regenerative outcome of the protocol, confirming the presence of newly formed bone tissue within the grafted sites. Furthermore, a significant clinical advantage observed was the concomitant formation of keratinized gingiva (Figure 5), which obviates the need for a secondary soft tissue grafting procedure that is often required with alternative techniques, thereby reducing patient morbidity and potentially mitigating long-term risks such as peri-implantitis [35,36,37]. It is important to note that while these histological findings are confirmatory, the analysis in this study was inherently qualitative, designed to verify bone formation; a quantitative histomorphometric assessment, though valuable, was not within the scope or feasibility of the present study design.

It is important to acknowledge that the 8-month follow-up period in this study primarily assesses the regenerative phase under the barrier. While the results demonstrate successful bone formation and keratinized tissue generation at 10 months, longer-term evaluation including prosthetic loading and implant stability at 12–24 months would provide more comprehensive evidence of the technique’s clinical efficacy and stability.

Methodologically, the use of both linear mixed-effects models and patient-level sensitivity analyses strengthens the statistical validity of our findings. The consistent results across different analytical approaches, while accounting for the clustered data structure, provide robust evidence for the bone regeneration achieved with this technique.

In summary, while titanium barriers appear to offer potential advantages over conventional techniques in terms of reduced invasiveness and operative simplicity by obviating the need for autologous bone grafting and facilitating soft tissue management, the descriptive nature of the present prospective case series, its limited sample size, and uncontrolled design require a cautious interpretation of the findings. Consequently, direct comparisons with other guided bone regeneration techniques cannot be made, and future controlled studies with larger cohorts are needed to evaluate relative effectiveness, determine long-term benefits, and confirm the consistency and reproducibility of the observed outcomes [38].

## 5. Limitations

This study has several limitations that should be considered when interpreting the results. First, it was designed as a prospective case series without a control group; therefore, it represents a lower level of clinical evidence and does not allow direct comparisons with other guided bone regeneration techniques, nor does it support conclusions regarding equivalence or superiority. Second, the exploratory, uncontrolled design and small sample size limit the generalizability of the findings; no a priori power calculation was performed, and together with the limited number of clusters (13 patients), this may reduce the precision of estimates and render mixed-model variance components unstable, so *p*-values and confidence intervals should be interpreted cautiously within this hypothesis-generating framework. Third, the evaluation focuses on the regenerative phase prior to functional loading; as such, the study does not provide data on implant survival, peri-implant tissue stability, or bone behavior under load. Finally, while the observed low complication rate is encouraging, it should be interpreted with caution in light of the small, uncontrolled cohort and the pre-loading follow-up; no inferences should be made regarding reduced exposure risk or superiority over other methods in the absence of controlled comparative data. Longer-term follow-up under functional loading (12–24 months) and controlled studies with larger cohorts—including quantitative histomorphometry—are needed to assess effectiveness, stability, and generalizability of this protocol.

## 6. Conclusions

This prospective case series provides initial evidence supporting the feasibility of a modified guided bone regeneration (GBR) protocol using customized titanium barriers with a window design, combined with an autologous blood clot and tricalcium phosphate. Within the constraints of this study design, significant bone gains were achieved in both horizontal and vertical dimensions (mean horizontal gain: 4.50 mm; mean vertical gain: 4.40 mm), suggesting the potential of this approach to restore bone volume in complex alveolar defects. Histological analysis confirmed the presence of newly formed, well-vascularized bone with active osteoblasts and keratinized gingiva, without signs of inflammatory response, findings that are consistent with a favorable regenerative process.

Importantly, the present report is explicitly limited to the evaluation of the regenerative phase prior to functional loading. Although implant placement was successfully performed at all regenerated sites according to the predefined protocol, this study does not provide data on implant survival, prosthetic loading, or long-term clinical performance. Consequently, the results should be interpreted strictly within the context of early regenerative outcomes.

From a methodological and clinical perspective, this protocol demonstrated potential advantages, including avoidance of autologous bone harvesting and simplified soft-tissue management by eliminating the need for primary wound closure. In addition, the window design allowed continuous clinical monitoring during healing. Together, these features suggest that this approach may represent a viable alternative within the spectrum of GBR techniques, particularly in moderate to severe defects.

Nevertheless, despite the use of rigorous statistical methods that accounted for the clustered data structure, the limitations of this study—including its small sample size, uncontrolled design, and qualitative histological assessment—necessitate a cautious interpretation of the findings. Future controlled studies with larger cohorts, quantitative histomorphometric analyses, and long-term follow-up under functional loading are required to further evaluate the efficacy, consistency, and clinical value of this protocol.

## Figures and Tables

**Table 1:** Descriptive values of the pre- and postoperative measurements.

Variable	Mean (mm)	SD (mm)	Median (mm)	Range (mm)	IQR (mm)
Horizontal preoperative	3.56	1.38	3.17	1.42–8.70	2.18
Horizontal postoperative	8.06	1.96	7.88	5.10–12.90	2.62
Horizontal gain	4.5	2.02	4.26	1.27–8.63	2.03
Vertical preoperative	10.68	6.17	9.69	1.50–24.10	10.25
Vertical postoperative	15.09	5.89	13.97	6.15–26.63	13.19
Vertical gain	4.4	2.82	3.68	0.10–10.60	3.99

**Table 2:** Comparative outcomes between the customized titanium occlusive barrier technique and conventional GBR approaches.

Technique/Workflow	Representative Sources	Horizontal Bone Gain (mm)	Vertical Bone Gain (mm)	Complication Profile	Autologous Bone Required	Primary Closure Required	Salient Notes
Present cohort: Customized Ti occlusive barrier (window) + autologous blood clot + β-TCP	This study	4.50 ± 2.02	4.40 ± 2.82	Overall per-site: 7.1% (3.6% screw loosening; 3.6% window detachment); 0% exposure; 0% infection	No	No	Keratinized mucosa formed in all sites; barrier removed at ~6 months; implants placed at ~10 months.
Khoury technique (autogenous block grafts, tunnel approach)	Khoury & Hanser 2022 [9]; Sánchez-Sánchez 2021 [8]	~5.0–5.6	≥7.0	Donor site morbidity; exposure/dehiscence risk varies	Yes	Yes	High volumetric stability; surgical complexity and second surgical site required.
Urban protocol (vertical ridge augmentation; particulate graft + rigid mesh/membrane)	Urban 2023 [5] (review)	~5.0	4–7	Exposure rates often 10–20%; infection uncommon	Yes	Yes	Emphasis on PASS principles and tension-free closure; robust vertical outcomes with experienced operators.
Membrane-based GBR (resorbable or non-resorbable; biomaterials)	Mizraji 2023 [7] (overview)	3–5	2–5	Exposure/dehiscence ~5–25%; infection low	Yes	Yes	Widely adopted; outcomes depend on defect severity, membrane type, and flap management.
Customized CAD/CAM titanium meshes (closed designs)	Chiapasco 2021 [15]; Nan 2023 [31]; Ronda 2024 [10]; Cucchi 2025 [28]	4–6	3–8	Exposure rates ~10–20%; hardware issues occasional	Yes	Yes	Digital fit improves space maintenance; generally requires primary closure and flap release.

## Data Availability

The data that support the findings of this study are available on request from the corresponding author. The data are not publicly available due to their containing information that could compromise the privacy of research participants. Specifically, anonymized quantitative data are available subject to ethical approval, while the underlying clinical imaging (CBCT) and 3D model (STL) data are subject to further restrictions. No custom software code was generated.

## Supplementary Materials

The following supporting information can be downloaded at: https://www.mdpi.com/article/10.3390/biomimetics11020149/s1, Document S1: STROBE Statement; Document S2: PRISMA-S-Informed Literature Search Strategy for Narrative Contextualization. Figure S1: Simplified schematic of the customized Ti6Al4V occlusive barrier featuring an integrated window.

## Abbreviations

The following abbreviations are used in this manuscript:

β-TCP	β-Tricalcium Phosphate
CAD	Computer-Aided Design
CAM	Computer-Aided Manufacturing
CBCT	Cone Beam Computed Tomography
GBR	Guided Bone Regeneration
STL	Stereolithography
SSS	Sterile Saline Solution
DICOM	Digital Imaging and Communication in Medicine
HU	Hounsfield Unit
ICP	Iterative Closest Point
TRE	Target Registration Error
ICC	Intraclass Correlation Coefficient
SEM	Standard Error of Measurement
