# Supplementary material for: Prospective Clinical Evaluation of Customized Titanium Occlusive Barriers with Window Modification for Guided Bone Regeneration: Radiographic and Histological Outcomes
Source: Biomimetics (Basel). 2026 Feb 17;11(2):149. doi: 10.3390/biomimetics11020149 (PMC12937595; doi:10.3390/biomimetics11020149)
Supplement: Supplementary file 1 [file biomimetics-11-00149-s001.zip › Document S2.pdf]

Supplementary Material S2: PRISMA-S-Informed Literature Search Strategy for Narrative Contextualization

1.Introduction and Rationale

Tooth extraction initiates alveolar bone resorption, which can compromise dental implant rehabilitation, making socket preservation and Guided Bone Regeneration (GBR) techniques critical for restoring bone volume [1,2,5,6]. The advent of CAD/CAM technology has improved GBR predictability by enabling the fabrication of customized titanium barriers that provide superior space maintenance and stability for bone healing [7,8,20-23]. The success of these techniques relies on adhering to biological principles, such as the PASS criteria, and on utilizing biomaterials like tricalcium phosphate to stabilize the blood clot and provide an osteoconductive scaffold [9,14-18]. Furthermore, the management of soft tissues, including the preservation of keratinized mucosa, is essential for long-term implant success [19].

To rigorously contextualize the findings of the present study—which evaluates a customized titanium barrier with a modified window design—within this existing scientific landscape, a structured literature search was conducted. The objective was to transparently identify key publications for comparing clinical outcomes, explaining biological rationales, and discussing the relative advantages and limitations of the presented technique against established protocols.

2. Search Strategy and Selection Process

A targeted literature search was conducted to identify key publications on established GBR techniques, biomaterials, biological principles, and clinical outcomes.

- **Information Sources:** The primary database consulted was **PubMed/MEDLINE**. Supplementary searches were performed using **Google Scholar** to ensure coverage of recently published or highly relevant articles not yet indexed in MEDLINE.
- **Search Date:** The final search was conducted on [Insert Date, e.g., "February 20, 2025"].
- **Eligibility Criteria:**
    - **Population/Concept:** Studies on humans or animal models focusing on alveolar bone regeneration.

- **Intervention/Context:** Techniques including, but not limited to, the use of titanium meshes/barriers, autogenous bone grafts, tricalcium phosphate (TCP) biomaterials, and the principles of wound healing in GBR.
- **Comparator:** Established protocols such as the Khoury technique, Urban technique, and other major GBR approaches for vertical and horizontal ridge augmentation.
- **Outcomes:** Primary outcomes of interest were quantitative bone gain (horizontal and vertical), histological evidence of regeneration, and reported complications. For biological rationale papers, the focus was on mechanisms of action (e.g., osteoconduction, epithelial migration).
- **Study Types:** Seminal clinical studies, randomized controlled trials, systematic reviews, meta-analyses, and key opinion papers from high-impact journals were prioritized.

- **Search Syntax (PubMed):** A combination of the following MeSH terms and keywords was used, adapted for each specific concept:
  - ("Guided Bone Regeneration"[Mesh] OR "Alveolar Ridge Augmentation"[Mesh] OR "Bone Regeneration"[Mesh]) AND
  - ("Titanium"[Mesh] OR "Metal Mesh" OR "customized barrier") OR
  - ("Bone Transplantation"[Mesh] OR "Autografts" OR "Khoury technique" OR "Urban technique") OR
  - ("Tricalcium Phosphate"[Mesh] OR "Biomaterials"[Mesh]) OR
  - ("Wound Healing"[Mesh] OR "Epithelium"[Mesh] OR "PASS principles")
- **Study Selection:** The selection process was narrative and purposive rather tan exhaustive. The aim was to identify a representative body of high-quality, frequently cited literature to construct a fair and evidence-based discussion. Papers were selected based on their direct relevance to the comparative claims made in the manuscript (e.g., providing benchmark bone gain values for other techniques) and their authority in explaining the biological rationale behind the surgical protocol (e.g., the need to prevent epithelial ingrowth).

3. Limitations of the Search

This was not a systematic review intended to capture every relevant paper. Therefore, it is possible that some relevant studies were not identified. The purpose was to provide a rigorous and transparent foundation for the narrative comparison and biological justification presented in the discussion, not to perform a novel synthesis of all available evidence on GBR.

4. Included Studies and Rationale

The final set of cited references was chosen to serve specific roles in the manuscript's narrative:

- **Benchmarking Outcomes:** References [30] and [31] were selected as they provide quantitative, peer-reviewed data on bone gain from well-established GBR techniques (Khoury/Urban), allowing for a direct comparison with the outcomes of the present study.
- **Biological Rationale:** References [18] (PASS principles), [29] (epithelial exclusion), and [16,17] (TCP mechanism) were included as they are foundational or highly-cited studies that provide the scientific basis for the surgical and postoperative decisions made.
- **Technical Context:** References [8] and [28] (previous work by the authors) and [7,22] (studies on other titanium mesh systems) were included to contextualize the technical evolution and current state of the art for the specific intervention under investigation.

A full list of the references cited in the main manuscript is provided in its bibliography.
