# Supplementary material for: Prospective Clinical Evaluation of Customized Titanium Occlusive Barriers with Window Modification for Guided Bone Regeneration: Radiographic and Histological Outcomes
Source: Biomimetics (Basel). 2026 Feb 17;11(2):149. doi: 10.3390/biomimetics11020149 (PMC12937595; doi:10.3390/biomimetics11020149)
Supplement: Supplementary file 1 [file biomimetics-11-00149-s001.zip › Figure S1.pdf]

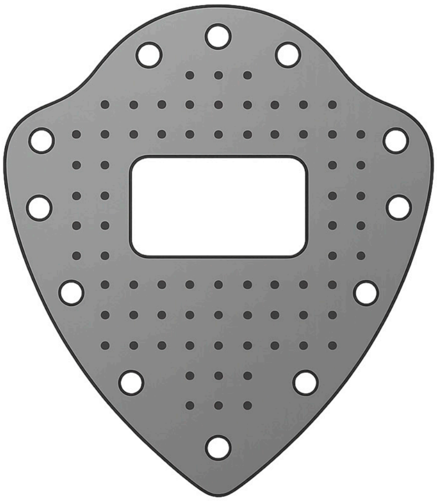

Figure S1. Simplified schematic of the customized Ti6Al4V occlusive barrier featuring an integrated window. The design illustrates:
(1) **Barrier body** (thickness ≈ 0.7 mm), digitally designed from CBCT-derived STL and manufactured via laser sintering;
(2) **Central window** for clinical access, irrigation, and protocolized de-epithelialization during healing;
(3) **Peripheral fixation** holes (Ø 1.5 mm) for screw anchorage ensuring stability over the defect;
(4) **Micro-perforation matrix** for weight reduction and potential tissue interface.
